# Drug use patterns and related factors among female sex workers in Iran in 2019–2020: results from Integrated Bio-Behavioral Surveillance-III (IBBS-III)

**DOI:** 10.1186/s13690-023-01143-x

**Published:** 2023-06-30

**Authors:** Bushra Zareie, Mohammad Aziz Rasouli, Mohammad Mehdi Gouya, Samaneh Akbarpour, Fatemeh Hadavandsiri, Elham Rezaei, Yousef Moradi, Ali Soltani, Ghobad Moradi

**Affiliations:** 1 Department of Epidemiology, School of Public Health, Hamadan University of Medical Sciences, Hamadan, Iran; 2 Clinical Research Development Unit, Kowsar Hospital, Kurdistan University of Medical Sciences, Sanandaj, Iran; 3 Social Determinants of Health Research Center, Research Institute for Health Development, Kurdistan University of Medical Sciences, Sanandaj, Iran; 4 Iranian Center for Communicable Diseases Control, Ministry of Health and Medical Education, Tehran, Iran; 5 Occupational Sleep Research Center, Baharloo Hospital, Tehran University of Medical Sciences, Tehran, Iran; 6 Cancer Research Center, Shahid Beheshti University of Medical Sciences, Tehran, Iran; 7 HIV/ STI Surveillance Research Center, and WHO Collaborating Center for HIV Surveillance, Kerman University of Medical Sviences, Kerman, Iran; 8 Department of Restorative Dentistry, Faculty of Dentistry, Kurdistan University of Medical Sciences, Sanandaj, Iran

**Keywords:** Female sex workers, Drug use, High risk behaviors, Bio-behavioral surveillance, Iran

## Abstract

**Introduction:**

Drug use is highly prevalent among female sex workers (FSWs). Some forms of drug use, such as injecting drug users (IDU), put them at greater risks for HIV and blood born disease (BBD). In this study, the pattern of drug use and its related factors among Iranian FSWs were investigated.

**Materials and methods:**

This cross-sectional study was performed based on the data of the integrated bio-behavioral surveillance-III (IBBS-III) on FSWs in 8 cities of Iran using the respondent-driven sampling (RDS) method conducting in 2019–2020. Of the 1515 FSWs participating in the IBBS-III study, 1,480 answered questions about drug use. To calculate the prevalence of drug use lifetime and in the past month, weighted analysis was used. Univariate and multivariate logistic regression was used to investigate the factors related to drug use.

**Results:**

The prevalence of lifetime drug use and the prevalence of current drug use (single and poly drug use) among FSWs were estimated to be 29.3% and 18.86%, respectively. According to multivariate regression analysis, the odds ratio (odds) of lifetime drug use showed a statistically significant association with lower education (AOR = 1.18; 95% CI: 1.07–1.3), being a direct sex worker (AOR = 1.77; 95% CI: 1.21–2.61), working in team houses or hangouts (AOR = 1.51; 95% CI: 1.10–2.06), a history of intentional abortion (AOR = 1.41; 95% CI: 1.07–1.87), condom use in the last sex (AOR = 1.61; 95% CI: 1.19–2.17), a history of imprisonment (AOR = 3.05; 95% CI: 2.25–4.14), HIV positive tests (AOR = 8.24; 95% CI: 1.66–40.9), alcohol use (AOR = 1.69; 95% CI: 1.29–2.29), and finding sexual clients in places such as parties, shopping malls, streets, and hotels, or by friends (AOR = 1.46; 95% CI: 1.01–2.12).

**Conclusion:**

Given that drug use among FSWs is about 14 times higher than that of the Iranian general population, it is imperative that drug reduction programs be integrated into service packages. Specifically, prevention programs should be prioritized for occasional drug users within this population as they are at a greater risk of developing drug use issues compared to the general population.

**Table:** 

Text box 1. Contributions to the literature
•It’s important to recognize that FSWs are a vulnerable population that faces a range of challenges and risks, and that addressing these challenges requires a comprehensive and multi-faceted approach that takes into account the complex interplay of physical, social, emotional, and economic factors that contribute to their vulnerability.
•Addressing the structural and systemic factors that perpetuate the vulnerability of FSWs may require changes to laws and policies that criminalize sex work and contribute to the marginalization of this population.
•Overall, addressing the challenges faced by FSWs requires a holistic and multi-faceted approach that takes into account the complex interplay of factors that contribute to their vulnerability. By addressing these factors, we can help to promote the health, well-being, and safety of this vulnerable population.

## Introduction

The high prevalence of drug use in FSWs has been reported in various international studies [1, 2]. FSWs may use drug as a mechanism to dull or numb themselves to future triggering encounters with clients. Most FSWs experience a wide range of physical, social, emotional, and economic situations which, individually or in combination, increase their vulnerability and put them at risk [3, 4]. Drug use among FSWs is a worldwide high-risk behavior in this group. Some FSWs may use it due to their dependence on drugs. Others may start taking it to meet the job and environmental challenges associated with sexual activities [5].

Studies show that deaths due to drug use occur faster among women than men, and in FSWs because of violence, deaths caused by drug use are increasing. One of the risk factors which has led to an increase in the diseases caused by drug use and alcohol use is unprotected sex [6]. Drug use and low or no condom use are high-risk behaviors which increase the prevalence of HIV in FSWs than the general population. Risks and harms of drug use are higher among FSWs, especially those with injecting drug users (IDU) and prisoners [5].

Estimates show that between 0.1% and 7.4% of the general population of women at reproductive ages in different regions are FSWs [7]. Also, about 57 to 90% of FSWs are involved in IDU, and between 46 and 96% of them are addicted to illegal drugs [8]. In Iran, drug use is also recognized as a common high-risk behavior among FSWs [9].

It is difficult to investigate drug use and FSWs because both activities are illegal and highly stigmatized. As a result, obtaining accurate information is difficult in this regard. Obtaining information about FSWs and drug use in this group increases our understanding of this group’s needs and helps to design appropriate interventions [10, 11]. Similar to other countries, there are different types of FSWs in Iran. The majority of FSWs in Iran engage in sexual activities in small groups and as an individual business but some are associated with homes and places known as "illegal brothels" where they can meet clients [3]. However, experts believe that FSWs located on the street are the most dangerous subgroups, especially those who have a profile of multiple high-risk behaviors, including drug use, and should be targeted by a prevention program [12]. Due to the importance of drug use in FSWs and its individual and social consequences, such as increasing the prevalence of HIV in them and subsequently in the society, and the inadequacy of national studies and limitations of previous studies, the present study was conducted to investigate the prevalence of drug use and its related factors among FSWs in surveillance system with IBBS-III of HIV / AIDS in Iran.

## Materials and methods

### Study design, setting and participants

This cross-sectional study was conducted using the data obtained through the integrated bio-behavioral surveillance-III (IBBS-III) survey among FSWs using respondent-driven sampling (RDS) in 2019–2020. The IBBS-III survey was conducted in eight large cities of Iran including Tehran, Tabriz, Shiraz, Mashhad, Kermanshah, Bandar Abbas, and Khorramabad.

Inclusion criteria included age 16 years or older, having sex (vaginal / anal) with more than one male client in the past year, living or working in the city, and giving initial consent to participate in the study. 1515 FSWs participated in the research.

### Data collection

Recruitment started with 4–9 seeds representing different groups of FSWs and data were collected face-to-face interview in the RDS centers. All eight cities used the same questionnaire containing different sections: demographic characteristics; history of sexual with clients; alcohol consumption and drug abuse; and discrimination and history of detention.

### Dependent variables

The dependent variables were the history of lifetime drug use as a binary variable (yes, no) and current drug use based on the frequency of drug use per person like no drug use, i.e. women who had not used any drug in the past six months, single drug use, i.e. women who used only one type of drug, and poly drug use, i.e. women who used more than one type of drugs. By drug, we mean Opium, heroin, Hashish, methamphetamine, Psychedelic drugs such as MDMA, Norgesic, Marijuana.Note that drug use in the last six months is considered as current use. Infrequent users were those who characterized their use as monthly or less; moderate users were those admitting to use on weekends or on no more than 2 days during any particular week; regular users used at least 3 days per week but no more than 5 days per week; daily users used nearly every day (6 or 7 days per week) [13].

### Covariates

The variables studied in this research included the age, marital status (single, married, divorced, concubine,^1^ widow, living with a partner), level of education (illiterate, elementary school, middle school, high school, diploma and academic), type of FSWs (direct (transactional sex as the main source of income), indirect (the main source of income is not transactional sex), age at the first vaginal or anal sex, age of first transactional sex, history of presence in team houses and hangouts to have sex with clients or find a client (yes, no), the main way of finding customers (team houses / hangouts, referrers (owners and pimps), virtual sex (via a mobile, internet and social networks) and others (parties, shopping malls, streets, parks, hotels and inns, and public transportation places, or friends)), history of anal sex (yes, no), history of group sex (yes, no), history of intentional abortion (yes, no), number of clients in the past month (1, 2–5, 6 clients and more), condom use in the last vaginal, anal or oral sex (yes, no), history of detention or imprisonment (yes, no), result of HIV serology tests (positive, negative) and lifetime alcohol use (yes, no).

### Data entry and analysis

In this study, the data of all cities were merged after examining the possibility of errors in entering the data into the software and after cleaning. Finally, 1480 women who answered the questions related to drug use were analyzed. Descriptive analysis included weighted mean ± standard deviation (SD) or number (adjusted percentage) calculated based on RDS-II method and Bootstraps #1000. *P*-value in the contingency tables was assigned to the weighted frequencies calculated by RDS software not crude frequencies. The *P*-value obtained for qualitative and quantitative variables was based on Pearson's Chi-squared and t-test, respectively. We decided to consider lifetime use in a multivariate model, because we believe that any woman who is FSW and uses drugs at least once has the potential for risky behavior. To identify the factors associated with drug use, all the variables with a *P* < 0.05 at the univariable model were included in the multivariable analysis. Univariate and multivariate logistic regression was performed using non-weighted analysis based on the suggestion in the latest article. Stata software (version 14), RDS Analyze and R (version 4.1.2) were used to perform all analyzes. (For more information on the RDS software settings used about seeds, refer to [14]).

## Results

In this study, 1480 FSWs with a mean (standard deviation) (SD) age of 35.76 years (9.15) were evaluated. The prevalence of lifetime drug use and the prevalence of current drug use (single or poly drug use) among FSWs were estimated to be 29.3% and 18.86%, respectively. The mean (SD) age of onset of drug use in FSWs was 23.96 (7.69) years.

The results showed that the highest prevalence of lifetime drug use (33.27%) was in the age group of 41–50 years. Also, the prevalence of lifetime drug use was higher in concubine women. Single and poly drug use in widows and concubines were reported 12.85% and 17.24%, respectively. In this study, the prevalence of lifetime drug use in women living with a partner was reported 47.92%. Also, the prevalence of lifetime drug use in terms of the education levels was 37.18% in illiterate people. In terms of FSWs’ types, the prevalence of lifetime drug use was higher in women who was a direct sex worker (31.25%) (Table 1).

**Table 1:** Demographic characteristics by lifetime drug use and pattern of current drug use among FSWs in Iran during 2019–2020

Variables	*N* = 1480	History of lifetime drug use, N (weighted %)			Current drug use N (weighted %)			
		No	Yes	*P*-value	No	Single drug use	Poly-drug use	*P*-value
		*N* = 971 (70.7%)	*N* = 509 (29.3%)		*N* = 1171 (81.15%)	*N* = 132 (9.39%)	*N* = 177 (9.47%)	
Prevalence		70.7 (66.30–75.12)	29.3 (24.9–33.71)	-		9.39 (6.70–12.07)	9.47 (6.19–12.47)	**-**
Age mean ± SD	35.76 (9.15)	35.76 (9.05)	35.97 (9.41)	0.676	35.59 (9.07)	36.85 (9.89)	36.16 (9.11)	**0.840**
Age group								
≤ 30 years	468 (31.76)	315 (70.83)	153 (29.17)	0.249	367 (81.52)	47 (8.49)	54 (9.98)	0.478
31–40 years	599 (40.23)	395 (73.01)	204 (26.99)		487 (83.13)	44 (8.74)	68 (8.13)	
41–50 years	340 (22.88)	211 (66.77)	129 (33.23)		261 (77.61)	31 (11.21)	48 (11.17)	
≥ 51 years	73 (5.12)	50 (69.46)	23 (30.54)		56 (79.01)	10 (11.83)	7 (9.17)	
Marital status								
Single	146 (10.46)	92 (68.13)	54 (31.87)	0.002	117 (83.41)	14 (10.00)	15 (6.59)	0.002
Married	315 (24.94)	213 (71.52)	102 (28.48)		252 (78.30)	35 (9.90)	28 (11.79)	
Divorced	676 (46.05)	462 (74.78)	214 (25.22)		553 (85.29)	53 (8.30)	70 (6.41)	
Concubine	172 (8.13)	109 (61.37)	63 (38.63)		132 (73.77)	10 (8.98)	30 (17.24)	
Widow	117 (8.19)	69 (63.59)	48 (36.41)		82 (73.24)	12 (12.85)	23 (13.91)	
Living with partner	45 (2.23)	20 (52.08)	25 (47.92)		29 (77.07)	8 (13.70)	8 (9.23)	
Education								
Illiterate	109 (8.27)	59 (62.82)	50 (37.18)	0.011	73 (72.27)	14 (13.18)	22 (14.56)	0.066
Elementary	302 (21.69)	186 (68.49)	116 (31.51)		225 (79.61)	36 (11.92)	41 (8.47)	
Middle school	357 (25.07)	212 (67.47)	145 (32.53)		273 (80.06)	34 (8.98)	50 (10.96)	
High school	173 (10.89)	115 (69.69)	58 (30.31)		137 (79.33)	16 (11.10)	20 (9.57)	
Diploma	381 (24.10)	289 (76.30)	92 (23.70)		330 (85.40)	22 (6.90)	29 (7.70)	
Academic	157 (9.96)	109 (77.32)	48 (22.68)		132 (86.00)	10 (6.04)	15 (7.95)	
Type of FSWs								
Direct sex worker	1153 (79.90)	742 (68.75)	411 (31.25)	0.004	906 (80.33)	110 (10.38)	137 (9.29)	0.558
Indirect sex worker	233 (20.10)	177 (78.00)	56 (22.00)		192 (82.03)	20 (8.16)	21 (9.81)	

The results showed that the mean (SD) age of FSWs at the first sexual relation and the first transactional sex in individuals with lifetime drug use was 17.03 (4.25) and 26 (8.49) years, respectively, and the mean age of first transactional sex in FSWs showed a statistically significant difference for both outcomes. Also, the prevalence of lifetime drug use was reported in 39.06% of people who were in team houses and the prevalence of single and poly drug use in FSWs was 13.51% and 12.45%, respectively. The highest prevalence of single and poly drug use was reported 12.49% (in FSWs with 6 or more customers) and 14.10% (in FSWs with ones with 2–5 clients) respectively. The prevalence of drug use was higher in FSWs with a history of abortion, and imprisonment, with an HIV positive test, who had used a condom in their last sex, and who alcohol use and found customers in ways other than hangouts and pimps while they showed a significant association (Table 2). 273 (with a weighted percentage of 89%) of the FSWs who used drugs in the last month had a history of using drugs during their sexual activities in the last month. Among FSWs who have had lifetime drug use (with the weight percentage corresponding to the number), 35 (6.51%) of the FSWs are moderate users, 25 (4.14%) regular users, 236 (51.71%) daily users. The rest of the FSWs, 213(37.65%) are either infrequent drug users or did not answer the question.

**Table 2:** Behavioral characteristics, Sexual risk factors by history of lifetime drug use and pattern of current drug use among FSWs in Iran during 2019–2020

Variables	*N* = 1480	History of lifetime drug use N (weighted %)			Current drug use			
					N (weighted %)			
		No	Yes	*P*-value	No	Single drug use *N* = 132	Poly-drug use *N* = 177	*P*-value
		*N* = 971	*N* = 509		*N* = 1171			
Age of first sex contact mean ± SD		17.42 (4.28)	17.03 (4.25)	0.098	17.46 (4.37)	16.22 (3.49)	17.08 (4.02)	0.452
Age of first transactional sex mean ± SD		27.94 (8.54)	26.60 (8.49)	0.006	27.74 (8.49)	27.81(10.44)	25.71 (6.55)	0.003
History of worked in team houses/hangouts								
Yes	674 (32.14)	397 (60.94)	277 (39.06)	< 0.001	504 (74.04)	60 (13.51)	110 (12.45)	< 0.001
No	790 (67.86)	561 (75.43)	229 (24.57)		652 (84.41)	71 (7.48)	67 (8.11)	
Group sex								
Yes	402 (16.21)	250 (68.93)	152 (31.07)	0.636	314 (80.90)	35 (10.64)	53 (8.45)	0.687
No	1050 (83.79)	697 (70.73)	353 (29.27)		829 (80.96)	97 (9.26)	124 (9.78)	
Client number (past month)								
1	79 (7.15)	52 (69.35)	27 (30.65)	0.396	63 (79.82)	8 (10.54)	8 (9.65)	< 0.001
2–5	480 (38.44)	330 (70.11)	150 (29.89)		374 (78.58)	35 (7.32)	71 (14.10)	
≥ 6	748 (48.42)	484 (72.28)	264 (27.72)		596 (81.80)	83 (12.49)	69 (5.71)	
Don’t know	106 (5.98)	59 (63.37)	47 (36.63)		81 (86.63)	4 (4.40)	21 (8.97)	
History of anal sex in lifetime								
Yes	782 (43.45)	497 (69.32)	285 (30.68)	0.35	618 (81.67)	61 (8.63)	103 (9.70)	0.706
No	658 (56.55)	441 (71.75)	217 (28.25)		518 (80.79)	69 (9.92)	71 (9.29)	
History of intentional abortion								
Yes	582 (37.22)	348 (65.26)	234 (34.74)	0.001	450 (79.05)	51 (9.88)	81 (11.07)	0.182
No	775 (62.78)	533 (74.01)	242 (25.99)		622 (82.91)	70 (8.81)	83 (8.28)	
Condom use in the last sex								
Yes	1021 (70.71)	703 (73.24)	318 (26.76)	0.001	824 (82.19)	97 (9.97)	100 (7.84)	0.008
No	452 (29.29)	263 (64.59)	189 (35.41)		342 (78.88)	35 (8.22)	75 (12.90)	
History of arrest or prison								
Yes	423 (21.81)	170 (37.82)	253 (62.18)	< 0.001	259 (56.96)	53 (15.80)	111 (27.23)	< 0.001
No	984 (78.19)	740 (79.55)	244 (20.45)		845 (87.62)	76 (7.65)	63 (4.73)	
Violent sex								
Yes	590 (29.99)	341 (60.91)	249 (39.09)	< 0.001	441 (75.06)	56 (11.17)	93 (13.77)	< 0.001
No	885 (70.01)	626 (74.75)	259 (25.25)		726 (83.66)	76 (8.67)	83 (7.67)	
HIV sero-status								
Negative	1456 (98.37)	963 (71.44)	493 (28.56)	< 0.001	1156 (81.38)	129 (9.35)	171 (9.26)	0.104
Positive	24 (1.63)	8 (26.37)	16 (73.63)		15 (66.89)	3 (11.42)	6 (21.69)	
Alcohol use								
Yes	897 (53.24)	533 (65.64)	364 (34.36)	< 0.001	694 (79.83)	83 (9.42)	120 (10.74)	0.271
No	547 (46.76)	412 (75.58)	135 (24.42)		449 (82.21)	46 (9.60)	52 (8.20)	
The main way to find clients								
Hangout	143 (5.93)	99 (69.08)	44 (30.92)	0.003	112 (76.10)	11 (12.43)	20 (11.47)	< 0.001
Pimp	328 (21.27)	214 (69.17)	114 (30.83)		258 (79.02)	42 (15.59)	28 (5.39)	
Cyberspace (phone, internet)	264 (18.45)	198 (79.93)	66 (20.07)		232 (88.69)	16 (7.25)	16 (4.06)	
Others (party, streets, friends, hotel, etc.)	733 (54.35)	454 (68.33)	279 (31.67)		561 (79.86)	62 (7.33)	110 (12.81)	

Based on multivariate regression analysis, the odds of drug use in lifetime increase with decreasing education from the academic level compared to the lower levels (AOR = 1.18; 95% CI: 1.07–1.3), and who were direct sex workers (AOR = 1.77; 95% CI: 1.21–2.61). Work in team houses or hangouts showed a significant association with lifetime drug use (AOR = 1.51; 95% CI: 1.10–2.06). Also lifetime drug use showed a statistically significant association with a history of intentional abortion (AOR = 1.41; 95% CI: 1.07–1.87), condom use in the last sex (AOR = 1.61; 95% CI: 1.19–2.17), a history of imprisonment (AOR = 3.05; 95% CI: 2.25–4.14), an HIV sero- status positive (AOR = 8.24; 95% CI: 1.66–40.9), alcohol use (AOR = 1.69; 95% CI: 1.29–2.29), and finding a sex client in parties, shopping centers, streets, and hotels or by friends (AOR = 1.46; 95% CI: 1.01–2.12) (Table 3).

**Table 3:** Factors associated with History of lifetime drug use among FSWs in Iran 2019–2020 using logistic regression

	**History of lifetime drug use**			
**Variables**	**Crude OR (95% CI)**	***P*-value**	**Adjusted OR**	***P*-value**
Married				
Marital status	Ref		Ref	
Single	1.23 (0.81, 1.85)	0.331	1.37 (0.82, 2.31)	0.230
Divorced	0.97 (0.73, 1.29)	0.820	0.84 (0.59, 1.21)	0.353
Concubine	1.21 (0.82, 1.78)	0.344	0.62 (0.38, 1.01)	0.056
Widow	1.45 (0.94, 2.25)	0.094	1.28 (0.73, 2.24)	0.387
Living with partner	2.61 (1.39, 4.92)	0.003	2.22 (0.94, 5.26)	0.069
Education level^a^	1.20 (1.12, 1.29)	< 0.001	1.18 (1.07, 1.30)	0.001
Type of FSWs				
Indirect sex worker	Ref		Ref	
Direct sex worker	1.75 (1.27, 2.42)	0.001	1.77 (1.21, 2.61)	0.004
Age of first transactional sex	0.98 (0.97, 0.99)	0.008	1.00 (0.98, 1.01)	0.586
History of worked in team houses/hangouts				
No	Ref		Ref	
Yes	1.71 (1.38, 2.12)	< 0.001	1.51 (1.10, 2.06)	0.010
History of intentional abortion				
No	Ref		Ref	
Yes	1.48 (1.18, 1.85)	0.001	1.41 (1.07, 1.87)	0.014
Using of condom in last sex (Vaginal, anal or oral)				
No	Ref		Ref	
Yes	1.59 (1.26, 2.00)	< 0.001	1.61 (1.19, 2.17)	0.002
History of arrest or prison				
No	Ref		Ref	
Yes	4.51 (3.54, 5.75)	< 0.001	3.05 (2.25, 4.14)	< 0.001
Violent sex				
No	Ref		Ref	
Yes	1.76 (1.42, 2.20)	< 0.001	0.99 (0.73, 1.34)	0.952
HIV sero-status				
Negative	Ref		Ref	
Positive	3.91 (1.66, 9.19)	0.002	8.24 (1.66, 40.90)	0.010
Alcohol use				
No	Ref		Ref	
Yes	2.08 (1.65, 2.64)	< 0.001	1.69 (1.24, 2.29)	0.001
The main way to find clients Cyberspace (phone, internet)	Ref		Ref	
Hangout	1.33 (0.85, 2.09)	0.212	0.60 (0.33, 1.08)	0.089
Pimp	1.60 (1.12, 2.29)	0.011	1.03 (0.67, 1.59)	0.888
Others (party, shopping center, streets, friends, hotel, etc.)	1.84 (1.34, 2.53)	< 0.001	1.46 (1.01, 2.12)	0.047

^a^The level of *education* was coded as the highest level (basic level) to the lowest level

## Discussion

The results of the present study showed that the prevalence of lifetime drug use and the prevalence of current drug use (single or poly drug use) among FSWs were estimated to be 29.3% and 18.86%, respectively. Also in multivariate logistic regression, education levels, types of FSWs (direct/ indirect sex worker), work in team houses / hangouts, a history of intentional abortion, condom use in the last sex, a history of arrest or prison, HIV positive tests, alcohol use and finding a customer through other ways than hangouts and pimps showed a significant association with lifetime drug use.

In the present study, the prevalence of lifetime drug use among FSWs was estimated to be 29.3% (95% CI: 29.4–33.65). This rate is much higher than the prevalence of drug use in the Iranian adult women, which has been reported to be about 1.2% [15, 16]. However, the prevalence of lifetime drug use in international studies is wide, ranging from 2.6% to 7.4% in China [12, 17], 25% in the region with a high prevalence of HIV in India, 34% in China-Myanmar border [18, 19], and more than 90% in Australia [8]. The results of the study by Roshanfekr et al. in Iran showed that the prevalence of non-IDU lifetime and in the last month drug use was 60.3% (95% CI: 51–84) and 47.2% (95% CI: 38–67) respectively [3] while the study of Shokouhi et al. in Iran reported the prevalence of drug use in the last month at 24.9% (95% CI: 16.1–36.4) [20]. In the study of Ahmadi et al. in Iran, the prevalence of IDU was reported to be more than 20% among FSWs [21]. The results of a meta-analysis on FSWs in Iran showed that the prevalence of IDU and non-IDU was 67.5% (95% CI: 2.09–10.73) and 56.94% (95% CI: 44.68–68.78), respectively [22]. The results of a BSS study in Iran showed that 24.9% of FSWs had reported drug use in the last month [22]. A high prevalence of lifetime drug use among FSWs was reported in Iran and in the provinces of Mazandaran (59%) [23], Fars (69.9%) [24] and Tehran (90.7%). The longer the period of FSWs, the higher the prevalence of drug use. For this reason, harm reduction programs of drug use among FSWs are recommended to be prioritized to improve their health status and reduce the prevalence of HIV/AIDS in the community.

The pattern of drug use also varies among FSWs. In the study by Tegang et al., 79% of FSWs was reported more than one type of drug use [25]. The results of other studies in Iran showed that having more clients, experiencing sexual violence or forced sex, instable housing or homelessness, and imprisonment were significantly associated with the likelihood of crystal methamphetamine use, single and poly drug use [20, 26]. Drug use may also lead to an increase in other high-risk sexual behaviors [27].

The results of the present study showed that there was no statistically significant association between the marital status and lifetime drug use among FSWs, which was consistent with the results of other studies [28, 29]. According to the study of Roshanfekr et al., the odds of using non-IDU among divorced FSWs was 2 times higher than that in single FSWs [3]. Temporary marriage can be an indicator of poor communication or family support, and low socioeconomic status among sex workers, which is associated with an increased risk of drug use [20].

The results of the present study showed that there was a significant association between low education levels and lifetime drug use, which was consistent with the results of other studies in Iran and China [12, 20, 21, 28]. However, there are inconsistent results which show that drug use is higher in people with higher education [29, 30]. Also, in previous studies, a significant association was observed between low levels of education as one of the important indicators of individuals’ socio-economic status and drug use [15]. Of course, people with lower education may report behavioral problems more than people with higher education [31].

The association between sexual activities in team homes and lifetime drug use was reported in this research. In the study of Shokouhi et al. in Iran, a higher frequency of drug use has been reported among FSWs with a larger number of sexual partners, which indicates that the impact of drug use is greater on high-risk behaviors [20].

The results of the present study and other ones showed that the prevalence of lifetime drug use was higher among HIV positive FSWs [20]. In the study by Lau et al., unprotected sex due to lack of condoms, making more money, and the demand for unprotected sex by clients were among the factors associated with using IDU or non-IDU and the increased incidence of HIV [32]. Access to HIV prevention, treatment, and care programs is difficult for many FSWs and people with IDU, especially in low- and middle-income countries. Poor coverage of services to this group as well as the lack of government support increase the disease prevalence in FSWs [33].

The association between a history of alcohol use and lifetime drug use among FSWs was reported in this study, which was consistent with the results of the study by Li et al. [34]. In the study by Medhi et al., the results showed that alcohol use was more common with drug use among FSWs, and a significant association was observed between alcohol use (at least once a week) and drug use among FSWs [18]. Low socioeconomic status, income, and education levels increase the susceptibility of FSWs to drug use [35]. Simultaneous alcohol and drug use is among the undesirable behaviors which require prevention programs to be changed.

The results of the study showed that the odds of history of intentional abortion was higher in lifetime drug use FSWs. The association between intentional abortion and drug use has been reported in other studies, which was consistent with the results of the present study [35, 36]. Relations with multiple sexual partners, less awareness of condom use, or less access to contraceptive methods lead to unwanted pregnancies and an increase in intentional abortion [37].

The results showed that the prevalence of lifetime drug use was higher in FSWs who found their clients through parties, shopping malls, streets, parks, hotels and inns, public transportation places, and friends. Clubs, cafes, team houses or hangouts are the main places for FSWs to find sexual partners, drug use and thus engage in risky behaviors [35].

There were several limitations in this RDS study, one of which was the nature of cross-sectional studies. In this type of study, we could only examine the existence or non-existence of the association between independent factors and the pattern of drug use and not its causality. Another limitation of this study was the non-random nature of the study samples. Random sampling is almost impossible in all hidden groups, but RDS sampling is its best alternative for hidden populations. One of the main limitations in the field of FSWs is that a consent letter for FSWs under 18 years of age must be obtained from the legal guardian of these people, while most of these people do not have a guardian or cannot be reached at all. This issue leads to this important group being ignored. A suggestion for future studies is to include a question in the questionnaire about how many years FSWs have been using drugs continuously.

## Conclusion

Given that drug use among FSWs is about 14 times higher than that of the Iranian general population, it is imperative that drug reduction programs be integrated into service packages. Specifically, prevention programs should be prioritized for occasional drug users within this population as they are at a greater risk of developing drug use issues compared to the general population.

## Data Availability

The data that support the findings of this study are available from the corresponding author but restrictions apply to the availability of these data, which were used under license for the current study, and so are not publicly available. Data are however available from the authors upon reasonable request and with permission of the corresponding author.

## Footnotes

1 Concubine is a term that refers to marriage for a short period of time, not for the rest of their life, and does not include a legal contract between couples who are considered lifelong partners.
